# Impact of Housing and Neighborhood on Depression Among Older Adults in the Health and Retirement Study

**DOI:** 10.1093/geroni/igab046.1517

**Published:** 2021-12-17

**Authors:** Rachel Wilkie, Margarita Osuna, Jennifer Ailshire

**Affiliations:** 1 Spatial Sciences Institute, USC, University of Southern California, California, United States; 2 USC, University of Southern California, California, United States; 3 University of Southern California, Los Angeles, California, United States

## Abstract

Prior research has suggested that poor neighborhood and housing conditions can lead to worse psychological wellbeing. Most studies examine either neighborhood or housing conditions, but not both. Since neighborhood and housing conditions may be correlated it raises the question of whether one is a proxy for the other. We use data from the 2006 and 2008 waves of the Health and Retirement Study to examine associations between perceived neighborhood and housing conditions in 2006 and depressive symptoms (CES-D 8) score in 2008. We find that worse housing conditions and neighborhood safety are associated with more depressive symptoms two years later, even when controlling for prior depressive symptoms. Furthermore, housing and neighborhood conditions are independently related to increased depression symptoms over time. Our research contributes to a deeper understanding of the relationship between home and neighborhood environments and psychological wellbeing in older adults.

